# Stress- and smoke free pregnancy study protocol: a randomized controlled trial of a personalized eHealth intervention including heart rate variability-biofeedback to support pregnant women quit smoking via stress reduction

**DOI:** 10.1186/s12889-021-10910-w

**Published:** 2021-05-12

**Authors:** Willeke van Dijk, Mirjam Oosterman, Imke Jansen, Wieke de Vente, Anja Huizink

**Affiliations:** 1 Department of Clinical, Neuro and Developmental Psychology, Amsterdam Public Health research institute, Vrije Universiteit Amsterdam, Van der Boechorststraat 7, 1081 BT Amsterdam, the Netherlands; 2 Department of Clinical Child and Family Studies, Faculty of Behavioural and Movement Sciences, Vrije Universiteit Amsterdam, Amsterdam, the Netherlands; 3 Research Institute of Child Development and Education, University of Amsterdam, Amsterdam, the Netherlands

**Keywords:** Smoking cessation, Stress reduction, HRV-biofeedback, eHealth, Pregnancy, Low SES, RCT

## Abstract

**Background:**

Maternal smoking and stress during pregnancy are associated with adverse health effects for women themselves and are risk factors for adverse developmental outcomes of the unborn child. Smoking and stress seem to be intertwined in various ways. First, the majority of smoking pregnant women is of lower socio-economic status, which is associated with higher levels of perceived stress. Second, smoking women often report to smoke because they feel stressed. Third, quitting smoking often increases perceived stress levels initially. Therefore, effective interventions are needed to support women with smoking cessation by reducing stress. The aim of this study is to test the effectiveness of an eHealth intervention on stress reduction and smoking cessation.

**Methods/design:**

The Stress- and Smoke Free Start of Life (SSFSL) study is a randomized controlled trial (RCT) comparing a personalized eHealth intervention with a control condition. Inclusion criteria for the women are: (1) > 18 years of age, (2) < 28 weeks pregnant at recruitment, (3) currently smoking. Consenting participants will be randomly assigned to the intervention or control group. Participants allocated to the intervention group will receive an 8-week intervention delivered on their smartphone. The application includes psycho-education on pregnancy, stress, and smoking (cessation); stress-management training consisting of Heart Rate Variability-biofeedback; and a personalized stop-smoking-plan. Participants in the control condition will be invited to visit a webpage with information on pregnancy, stress, and smoking (cessation). Study outcomes will be collected via online questionnaires, at four timepoints: pre-intervention (baseline; t0), post-intervention (8 weeks + 1 day after t0; t1), follow up at two weeks after birth (t2), and follow up at three months after birth (t3). The primary outcome measure is self-reported smoking cessation. Secondary outcomes include daily self-reported number of cigarettes smoked, perceived stress, pregnancy experience, birth outcomes, and negative affectivity scores of the baby. Moreover, the mediating effect of stress reduction on smoking cessation will be examined, and possible moderators will be tested.

**Discussion:**

If the eHealth intervention is effective in smoking cessation among pregnant smoking women, it can be implemented as a tool into the health care in the Netherlands.

**Trial registration:**

Netherlands Trial Register, ID: NL8156. Registered on 11 November 2019.

## Introduction

Worldwide, substantial numbers of women who smoke, continue to smoke during pregnancy. Recently estimated prevalence of smoking pregnant women in the European Region was 8.1%, which was the highest percentage when compared to the world average of 1.7% [1]. Even though prevalence rates of pregnant smoking women have been decreasing over the years [2], these numbers are yet of concern for several reasons. Maternal smoking has serious implications for the developing fetus and the mother. Maternal smoking increases the risk of miscarriage [3], premature delivery, lower birth weight [4], and congenital abnormalities [5]. Among the single newborns born in the Netherlands with small-for-gestational age, 17% of the cases was found to be related to maternal smoking during pregnancy [6]. Negative effects of prenatal maternal smoking are not only observed in the early years of the child, but have also been associated with negative health outcomes later in life, such as asthma [7] and obesity [8]. Moreover, maternal smoking during pregnancy has been linked to reduced academic achievement and decreased cognitive abilities of children [9, 10] and to neurobehavioral problems in childhood [11–13].

Despite the growing body of evidence on the negative consequences of prenatal smoking for child development and despite extensive efforts to reduce the prevalence of prenatal smoking, a relatively large group of expecting mothers continues to smoke during pregnancy [1]. Factors associated with continued smoking during pregnancy are socioeconomic status (SES), nicotine dependence, and stress experienced during pregnancy [14, 15]. In general, women of low SES experience socioeconomic inequalities when it comes to financial resources, stability in living situation, general health, including more stress-related health problems and unhealthy lifestyle habits such as smoking [16, 17]. Stress plays an important role in initiating smoking and maintenance of smoking [18, 19]. According to the stress-coping model, smoking causes a temporarily reduction of negative affect and an increase in positive affect, which can be an explanation for the use of smoking as a coping strategy in situations of high stress [20]. Neurobiological models of addiction suggest that both stress and nicotine exposure trigger stress and reward circuits in the brain explaining why stress can increase the urge to smoke [18]. Moreover, high levels of stress hinder changing unhealthy lifestyle behaviors, such as smoking [21]. Increased activation of brain stress systems are thought to reduce an individual’s ability to cope with extra stressors and may strengthen the effects of acute nicotine exposure during abstinence, thereby contributing to the increased vulnerability to relapse caused by stress during periods of abstinence [19]. Elevated stress levels may thus explain why expecting women may fail to quit smoking, because pregnancy is a critical life event. Indeed, during pregnancy, many women experience some additional levels of psychosocial and pregnancy-related stress [22], due to physical changes related to pregnancy, concerns about childbirth and parenting, or relationship difficulties. These extra pregnancy related stressors can thus make smoking cessation even more challenging.

While stress thus seems to be a maintaining factor of smoking, stress experienced during pregnancy itself has also been associated with adverse offspring outcomes. Research has shown that prenatal stress is associated with delayed motor and mental development, and temperamental variation in infants and older children [23–26]. In addition, pregnancy-specific anxiety predicted more difficult child temperament, as indicated by negative emotional reactivity, fearfulness and falling reactivity [27].

Thus, as prenatal stress and smoking are both related to negative child outcomes, and stress seems to play a role in the maintenance of smoking, interventions targeting both stress and smoking may be highly effective. Unfortunately, existing programs aimed at smoking cessation have been mostly ineffective with high relapse rates, specifically among vulnerable women with low SES and high levels of perceived stress [14, 28, 29]. An important element that is missing in most smoking cessation programs thus far, is stress management. This element seems essential for smoking cessation programs, especially those targeting low SES women, since effective stress-coping techniques may help women quit smoking during pregnancy and reduce long-term relapse risk. Investing in relapse prevention is important, because relapse within the first six months after birth is common, with a pooled mean proportion of women that have re-started smoking at 6 months of 43% [30].

A method that has shown to reduce stress is Heart Rate Variability-biofeedback (HRV-BF) [31]. HRV is determined by the variation in time between consecutive heartbeats as assessed by the interval between successive R-wave peaks in an electrocardiogram (ECG). Higher HRV has been associated with more favorable physical and mental health outcomes [32–34]. The aim of HRV-BF is to increase HRV and stimulate cardiac regulation [35]. HRV is influenced by physiological factors such as respiration, and respiratory sinus arrhythmia (RSA) is the component of HRV that reflects the variation in heart rate due to respiratory activity [35]. A mechanism that regulates cardiac activity is the baroreflex [36], which activity is triggered by changes in blood pressure in the body’s large blood vessels in order to achieve homeostasis [35]. Physiological activity, such as breathing, affect baroreflex functioning [34]. Through HRV-BF, people learn a method of paced breathing with visual feedback about their HRV in order to detect the frequency at which HRV is maximized. Maximized HRV causes increases in baroreflex amplitude, which can improve reflex efficiency and, in turn, can positively affect autonomic activity modulation [36].

Recent research of our team showed that HRV-BF training was effective in reducing feelings of stress in pregnant and non-pregnant women [37]. These results are in line with a meta-analysis, including community and clinical adult samples, reporting that HRV-BF was associated with a large reduction of self-reported stress and anxiety [38]. HRV-BF may be regarded as an effective stress-reduction intervention, and may be beneficial for pregnant women to support them quit smoking while suffering from stress.

During the last decades, more and more health care is being offered via the Internet, for instance by means of eHealth platforms [39, 40]. Benefits of eHealth are the high accessibility for a large group of people and the usually low cost it entails. Moreover, eHealth is unrestricted by time and place and might reach populations that otherwise do not ask for support, for instance as a result of possible stigmatization. As stigmatization is a common problem among pregnant smoking women [41], and considering the many benefits of eHealth for this group [42], an app-intervention to support pregnant women quit smoking is promising. A recent systematic review supports this, as results show that eHealth approaches were moderately effective on smoking abstinence in a general population of adult smokers [43]. They also emphasize the use of an interactive and tailored approach of smoking cessation interventions.

Considering the benefits of eHealth, we have developed an application including HRV-BF, targeting pregnant women who want to quit smoking. HRV-BF is easy to learn and maintain in daily life, and at the same time requires little efforts. Thus, HRV-BF could be highly appropriate for pregnant smoking women with low SES, who would like to quit smoking. In order to gain knowledge about the underlying mechanisms and conditional factors of treatment effectiveness, various factors that are associated with stress and smoking need to be studied. Factors that may affect the treatment effect obtained in stress reduction and smoking cessation are: pregnancy experience [21], pregnancy-related anxiety [44], self-efficacy regarding smoking cessation [45], a person’s motivation to quit smoking [46], and COVID-19 related stress [47]. Moreover, focusing on indirect treatment effects is also relevant in this transitional and developmental context. For example, factors associated with parenting, such as parenting self-efficacy [48–50] and the bond that the mother feels with her child [51, 52] may also increase when women experience less stress and quit smoking. Parenting factors such as parenting self-efficacy and a warm mother-child bond have been associated with positive effects on child development (e.g. [48, 53]).

### The current study

The current study will examine the effectiveness of a personalized 8-week lasting eHealth intervention “Together with Eva”, consisting of adapted parts of existing quit-smoking and stress-management interventions and an HRV-BF prototype application by means of a randomized controlled trial. “Together with Eva” includes psycho-education on stress, smoking (cessation), and pregnancy; a personalized stop-smoking plan; stress reducing exercises, comprising of HRV-biofeedback, and problem-solving techniques. Participants in the control group will receive access to a webpage developed for this study where they can read information comparable to the psycho-education of the intervention group. This information is however summarized and not interactive. Overall, the main research question of this study is the following:

1. Is the app intervention “Together with Eva” an effective intervention to support pregnant women to quit smoking during pregnancy and stay abstinent after giving birth? The primary outcome measure is self-reported smoking cessation. Secondary objectives of this study are to investigate whether;
2. the intervention is effective on secondary outcomes (i.e. smoking behavior, perceived stress, birth outcomes, negative affectivity, parenting self-efficacy, pregnancy-related anxiety, feelings of mother-child bonding, and COVID-19 related stress),
3. the intervention effect on smoking cessation is mediated by the reduction of perceived stress, and whether;
4. variables such as program usage and experience, self-efficacy regarding smoking cessation, intrinsic motivation for life style behavior change, and pregnancy experience moderate the treatment effect.

## Methods and analysis

### Trial design

**Fig. 1:**
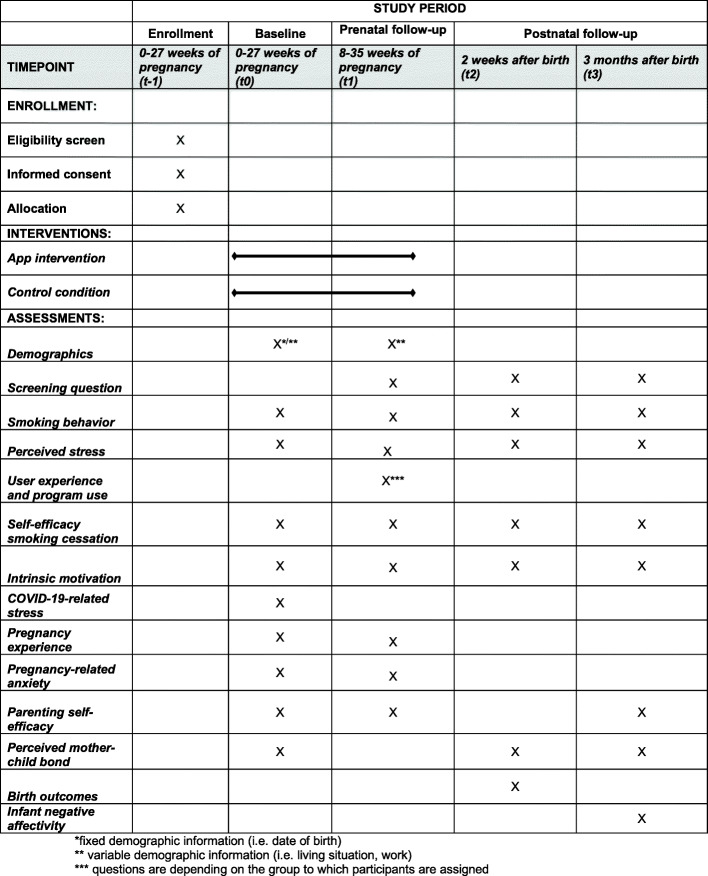
SPIRIT Flow Diagram of the Stress And Smoke Free Pregnancy Study

This study is a two-arm, parallel, single blinded randomized controlled trial (RCT) including four measurement waves: pre-intervention or baseline (t0), post-intervention (t1), two weeks following birth (t2) and three months after birth (t3). Pregnant, smoking women will be randomized to one of two groups: 1) personalized eHealth intervention (“Together with Eva”) on the smartphone followed for 8 weeks; or 2) active control condition which includes psycho-education on stress, smoking, and pregnancy through a website. The protocol is developed in accordance with the Standard Protocol Items: Recommendations for Interventional Trials guidelines (SPIRIT; Fig. 1). The trial is registered with the Netherlands Trial Register on November 112,019. The registration ID is NL8156. Ethical approval was obtained from the Medical Ethical Committee of the VU Medical Center (METc VUmc 2020/065/NL71342.029.20).

### Participants and recruitment procedure

Participants will be recruited through midwifery practices, specialized departments of hospitals in the Netherlands and through advertisements on reliable websites focusing on pregnancy over a 1.5-year period (July 2020–February 2022). Health care professionals will be offered flyers to hand out to potential participants. Additionally, they will receive an information sheet with the inclusion criteria, short sentences for study explanation, and a list of frequently asked questions so that they can easily explain the study to potential participants. Women are eligible for participation if they are older than 18 years, are less then 28 weeks pregnant (to enable intervention completion in view of the risk for premature birth in pregnant smokers), if they smoke on a regular basis (at least smoking 1 day/week), have an intention to quit smoking during pregnancy, and if they have sufficient command of the Dutch language. Women with severe psychiatric disorders, women who abuse other drugs (e.g. alcohol, cannabis, cocaine, and GHB), and who are participating in other intensive stop-smoking interventions will be excluded from participation. The focus of our recruitment will be on women with low SES. However, women with high SES are also allowed to participate.

### Sample size calculation

To ensure acceptable power for the main analysis of the intervention effect on smoking cessation at post-intervention, an a priori sample size calculation was performed by the program G*Power [54]. Using a power of 80% an alpha of 0.05 and an F-test, we need to include 160 participants (80 participants in each arm) in order to be able to detect a small difference (effect size of 0.20) between the intervention group and the control group on the primary outcome measure, smoking cessation at t1.

### Procedure

The participants will be informed about the study via obstetric practices, gynecologists, or general practitioners, but can also sign up for participation by own registration via a form on the project’s website. Pregnant women, who are visiting their health professional, will be given a brief explanation via a flyer. Women, who are willing to participate, will be asked to scan the QR-code on the flyer and will then be directed to an online registration form where they will fill in the contact details. This information will then be sent to a secured mailbox which can only be accessed by the main researcher. Subsequently, registered participants will be called by the researcher or research assistant who will explain the study in more detail. If participants are willing to participate they will be sent an information letter and consent form, which they will be asked to read, sign and send back. Then, women will be called to screen for exclusion criteria. Eligible women will receive an invitation email with a link to the first online questionnaire (t0). Participants will be assigned an identification number, which will be used throughout the study. To ensure confidentiality, the file including both the participants’ names and identification numbers will be saved with a password that can only be accessed by the principal researcher. After completion of the t0 questionnaire, participants will be randomized. This will be done by sending an email to an independent researcher who has access to the randomization list. The randomization list is created with blocks of 2, 4, and 6 participants, each containing equal numbers of participants allocated to the intervention and active control group, using an online randomization program. The independent researcher will send an email with information about the assigned group. Participants assigned to the intervention group receive an email with a link to download the application on their smartphones. The control group will be sent a link to the website with the psycho-education module, which they can access as frequently as they desire. After 8 weeks, participants will receive an invitation to complete the second questionnaire (t1). Two follow-up questionnaires will be sent, the first at two weeks after birth (t2) and the other at three months after birth (t3). Participants will receive 7.50 euros on their bank account for each completed questionnaire.

#### Intervention

The eHealth intervention “Together with Eva” of the current study consists of an 8 weeks lasting intervention offered on the participant’s smartphone. The intervention consists of an integration of an existing smoking cessation online intervention (www.mijnkoersroken.nl; developed by Trimbos institute) and an HRV-BF prototype application, Time2Breathe [55]. The smoking cessation intervention was adapted and personalized, and the HRV-BF prototype application was further developed by Aan Zee into an application including the actual HRV measurement and feedback Software Development Kit (SDK), developed by Happitech. Participants use the application at their own pace. The application consists of different components (see below), which will be supported by short instruction videos to explain the intended use.

##### Personalized stop-smoking plan

After registration in the app, participants will design a personalized stop-smoking plan in which the participant chooses a specific date on which she wants to quit smoking. Moreover, intrinsic motivation for the intended quitting attempt is enhanced by listing personal advantages of quitting and disadvantages of smoking. These lists will be referred to in the exercises in the app. Participants will also designate a person in their social network, who can be approached for help and support in times of craving. The phone number of this person will be saved in the app. When participants click on the button ‘I would like to call someone’, they will immediately be directed to call this number.

##### Smoking diary

During the 8 weeks of the intervention, participants will receive daily notifications to fill out their diary. In this diary, participants will report if and how many cigarettes they have smoked. This self-report on their smoking behavior will be used to enable positive feedback. For instance, if a woman has been able to refrain from smoking for a day, a week, and so on, she will receive a positive notification such as “Yes, keep it up!”. Participants can navigate through the diary, which gives a clear overview of the quitting process.

##### Personalized stress reduction training

Another element of the intervention is a personalized stress-reduction training. This training consists of two components: (a) HRV-BF, and (b) psycho-education including personalized and evidence-based problem-solving techniques.

##### HRV-bf

**Fig. 2:**
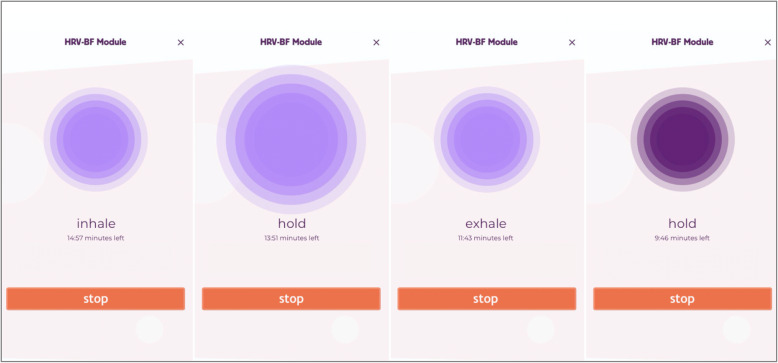
Screenshots of Instructions for Breathing in the HRV-BF Module of the Application

The HRV-BF module in the “Together with Eva”-app teaches the participant the technique of slow-paced diaphragmatic breathing through guided breathing exercises. Breathing in synchrony with the baroreflex, also called resonance frequency, results in a substantial increase in HRV [36]. In the first week of the intervention, participants are instructed to exercise slow diaphragmatic breathing, also called ‘belly-breathing’, without using their phones. From the second week of the intervention onwards, breathing exercises are guided by a breath pacer and visual feedback based on continuous HRV measurement developed by Happitech (Heart Rhythm SDK version 1 Happitech). HRV is measured through photo plethysmography (PPG) with use of the camera and flash on the mobile phone. Throughout the 8-week intervention period, the participant’s resonance frequency is assessed four times using a series of breathing exercises at the following fixed paces of 6.5, 6.0, 5.5, 5.0, 4.5 breaths/min, which is in accordance with resonance frequency protocols (e.g. [35]). The pace resulting in the highest frequency power peak in the low frequency (LF) power band (i.e., 0.04–0.15 Hz) relative to the total power, defined as the 0.02–0.5 Hz power band of HR data, is considered resonance frequency and is the advised breathing pace for the participant. During the regular breathing exercises, the participant is guided towards optimal breathing through the color of the breath pacer, which turns dark purple as the LF power peak increases (see Fig. 2). Panel 1 shows the instruction to breathe in; panel 2 instructs to hold the breath for 1 s; panel 3 instructs to breathe out; and panel 4 shows the adjustment of the color, becoming darker purple, when breathing more in synchrony with one’s baroreflex, which is the desired result of the breathing exercise. Breathing exercises are practiced twice a day at a time convenient for the participant. In the first week, breathing exercises last 5 min, whereas from the fourth week onwards, they last 15 min. After performing a breathing exercise, participants are asked about their experience, as indicated by a happy, neutral, or sad smiley, and whether they felt dizzy or nauseous during the exercise. The daily duration of the paced breathing with HRV-BF and the information about one’s experience will become visible in the diary.

Participants assigned to the intervention group will receive a phone call from the researcher or research assistant directly after randomization. The researcher will then instruct the participants to fill out the stop plan and will discuss when the participant is planning to use the app and do the breathing exercises. Participants are instructed to start using to app on that same day or the next day. Based on this schedule, researchers will have weekly phone calls with the participants to monitor the planning and, if necessary, adapt it for the following week.

##### Psycho-education on pregnancy, relaxation, and smoking cessation including personalized and evidence-based problem-solving techniques

A psycho-education module, called ‘Lessons’, offers participants written information about pregnancy, stress and relaxation, and smoking and smoking cessation. The lessons about pregnancy provide written information that matches with the gestational age of the unborn (estimated date of birth filled in at registration). In the lessons about stress and relaxation, participants can for instance read about what stress does to the body and mind, tips on stress management and how feelings of tension can be reduced, which is supported by evidence-based problem-solving exercises. With these problem-solving techniques, women can address their own problems or stressful situations, with guidance from the eHealth application. An important first step in this stress reduction is making a stress diary using notes written with pencil and paper, to identify stressful situations and problems. Next, women will be asked to make a clear difference between problems that can be solved and those that cannot be solved, but need to be accepted. Acceptance and tolerance for the unsolvable problems and associated emotions are boosted by building awareness that emotions related to these problems will pass. Then, active problem-solving techniques are built up in subsequent steps to deal with the potentially solvable problems: (1) the identification of a stressful situation or problem that causes stress that may be solvable, (2) the definition of one’s goal and how the problem hampers that goal, (3) finding solutions, also through seeking social support, (4) planning solutions, (5) implementing solutions, (6) taking stock: reviewing one’s situation again and see if there is a continuous need to deal with the problem more actively. In the lessons about smoking cessation, information about the effects of stopping smoking cigarettes on your body and mind is provided, and participants receive tips to refrain from smoking. Also, frequently asked questions about smoking and quitting will be discussed. New lessons will appear every week. If participants do not use the application as intended, they will be reminded by a text message, or a phone call if needed. Contact information of the researcher and of a person independent of the study is mentioned under ‘Contact’ so that the participants will always be able to ask questions if things are unclear or if they need help with exercises. Questions will be answered within 24 h on weekdays.

##### The craving-button and the smoked-button

A craving button ‘Help, I feel the urge to smoke’ is included on the home-screen of the app, which can be pressed when the participants expriences craving. When participants click this button, they will be asked several questions to find out what kind of information or exercise is desired to provide them with distraction and/or support to resist the sense of craving. Participants will either be recommended to talk to someone (i.e. social support that they have designated in their stop plan), to do something relaxing or distracting (e.g. a slow breathing exercise, going outside), or to read something (psycho-education modules). Furthermore, the button ‘Oops, I have smoked’ will help participants to deal with negative thoughts after they have smoked.

#### Control condition

The control condition consists of a website designed specifically for this study consisting of psycho-education on pregnancy, smoking during pregnancy, and stress. The information on the website is a summary of the psycho-education modules on stress, smoking and smoking cessation, and pregnancy included in the app-intervention. Since the website does not include exercises, it is less personalized, less intensive and interactive when compared to the intervention. This webpage can be visited as often as the woman desires and can be accessed on smartphone or computer.

### Outcome measures

At four different time points, women will receive online questionnaires. Each questionnaire will take approximately 15–20 min to complete. The first questionnaire is a baseline questionnaire (t0, before randomization) including the main outcomes and demographic information (i.e. date of birth, weeks of pregnancy, ethnicity, educational level, employment status, relationship status, smoking behavior partner, living conditions). Eight weeks and one day after group assignment, participants will receive the second questionnaire (t1), through which post-intervention measurements will take place. Follow-up questionnaires will be sent two weeks after birth (t2) and three months after birth (t3). In Fig. 1, an overview of the outcome measurements for each time point is depicted. Prior to t1, t2, and t3, a screening question will appear asking about the health of the baby. In case of pregnancy loss or if the baby died after birth, participants will be advised to quit the study participation. They will still receive the agreed compensation for the completed questionnaires.

#### Primary outcome

##### Smoking cessation

Smoking cessation and abstinence will be assessed using a self-report question ‘Do you smoke?’ (yes/no). If answered with ‘yes’, an additional question asking whether she smokes on a daily basis will indicate the frequency of the smoking behaviour.

#### Secondary outcomes

##### Smoking behavior

If participants indicate that they (still) smoke, they will be asked the number of cigarettes smoked per day. Thereby, it can be assessed whether the participant’s smoking behavior changed between the different time points.

##### Birth outcomes

Birth outcomes such as gender, birth date, gestational age and weight at birth will be assessed with self-constructed questions. Moreover, 1- and 5-min Apgar scores will be asked. These scores can be found in a booklet that women usually receive after delivery in the Netherlands. In case these scores are unknown to the women, consent will be asked to contact the midwifery practice in order to obtain these scores.

##### Infant temperament

Infant temperament will be measured at 3 months of age using the short version of the Infant Behavior Questionnaire (IBQ-R; [56]). From the total of 14 scales, the scales ‘Distress to Limitations’ and ‘Fear’, each consisting of 16 items, were used in this study. The mother will be asked to rate the frequency that her baby engaged in specific day-to-day behaviors during the previous week or two weeks on a scale from 1 (never) to 7 (always). Examples for items are ‘After sleeping, how often did the baby cry if someone doesn’t come within a few minutes?’ and ‘When introduced to an unfamiliar adult, how often did the baby refuse to go to the unfamiliar person?’. In a sample of Dutch mothers who reported on the behavior of their 4-month old babies, internal consistency of both the ‘Distress to Limitations’ and ‘Fear’ scale has been found to be good (*α* = .75 and .77, respectively; [57]).

##### Pregnancy-related anxiety

Pregnancy-related anxiety will be assessed using the PRAQ-R2 [58], a short version of the Pregnancy-Related Anxiety Questionnaire (PRAQ; [59]), suited for both women pregnant for the first time, as for women who already have children. The PRAQ-R2 consists of ten items divided into the three subscales (1) Fear of birth (3 items), (2) Worries about Bearing a Physically or Mentally Handicapped Child and complications during and after childbirth (4 items), and (3) Concern about own Appearance (3 items). Examples of items are ‘I am worried about the pain of contractions and the pain during delivery’ and ‘I am worried about the fact that I shall not regain my figure after delivery’, which can be answered on a 5-item Likert scale ranging from 1 (‘Does not apply to me at all’) to 5 (‘Does apply to me a lot’). Higher scores indicate more pregnancy anxiety. Internal consistency of the questionnaire for both nulliparous and parous women is acceptable (Cronbach’s *α* = 0.82–0.85; [58]).

##### Parenting self-efficacy

Parenting self-efficacy will be assessed by the Dutch version of the Maternal Self-efficacy in the Nurturing Role Questionnaire (SENR; [60]) during and after the pregnancy. The SENR has both a prenatal and postnatal version and both versions contain 16 situations regarding (expected, for the prenatal version) feelings of competences in taking care of a child, such as ‘I feel competent in my role as a parent’ and ‘I feel like I am not prepared for parenthood’, answered on a 7-point Likert scale ranging from 1 (Completely disagree) to 7 (Completely agree). A total score will be generated by summing up scores from each item, with higher scores indicating higher parental self-efficacy. Internal reliability has been found to be high (*α* = .86–.89; [61]).

#### Mediator

##### Experienced stress

Experienced stress will be measured using the Dutch, short version of the Perceived Stress Scale (PSS; [62, 63]). This scale consists of 10 items about subjective experiences of unpredictability, lack of control, and burden during the last month, e.g. ‘In the last month, how often have you been angered because of things that were outside of your control?’ Participants indicate on a 4-point scale from ‘never’ to ‘always’ to what extend the items apply to them. Higher scores indicate higher perceived stress. Items and answer categories have been adapted to make it more suitable for our target group. Previous studies reported acceptable to good internal consistency of the PSS (Cronbach’s ﻿α 0.78–0.91; [64]).

#### Moderators

##### User experience

User experience of both the application ‘Together with Eva’ and the webpage (https://www.fgb.vu.nl/nl/onderzoek/stress-en-rookvrij-zwanger) of the control condition will be assessed with several self-constructed questions, such as ‘How many times have you used the app/webpage’, answered by a 5-point Likert scale ranging from 0 (Not much/Never) to 4 (Every day). If they indicate that they have not used the application, they will be asked for the reason. Moreover, attractiveness and ease of use of the application will be assessed with 6 items from the Dutch translation of the User Experience Questionnaire (UEQ; [65]). Participants will answer the question ‘Please indicate what you think about the app’ and ‘Please indicate what you think about the breathing exercises’, answered on a scale from 1 (negative) to 7 (positive), with varying specifications such as ‘not interesting/ interesting’ or ‘unattractive/attractive’. Participants from the intervention group will also be asked if they were motivated to do a breathing exercise when they were stressed and whether they would recommend the application to a pregnant friend who would like to quit smoking.

##### Self-efficacy regarding smoking cessation

Self-efficacy regarding smoking cessation will be assessed by six items from a questionnaire developed by [66]. Participants are asked to indicate whether they are able to refrain from smoking if they find themselves in specific situations, for instance when they feel stressed or tense and when someone offers them a cigarette. Questions can be answered by 0 (never) to 3 (always). Higher scores indicate higher self-efficacy regarding smoking cessation.

##### Perceived mother-child bond

Mother-child bond will be measured using six items derived from a questionnaire developed for Generations^2^ study, which is based on the Treatment Self-Regulation Questionnaire (TSRQ; [67]). This questionnaire has a prenatal version asking about the future mother-child bond, and a postnatal version asking about the current bond of the mother and child. In the first question, the participant will be asked the extent to which she assumes a good relationship with her child is important, which can be answered by five categories ranging from 0 (does not apply to me at all) to 5 (does apply to me a lot). If the participant indicates that the relationship with her child is important to her (answer options 3–5), five items will appear asking about the reason for striving a good relationship. If the participant indicates that relationship with her child is not important (answering options 1–2), she will be asked whether she ever thinks about the relationship with her child. Higher scores indicate a stronger perceived mother-child bond. Internal consistency of the TSRQ was found to be acceptable [67].

##### Pregnancy experience

Pregnancy experience will be measured by the Dutch translation of the Pregnancy Experience Scale (PES; [68]), ‘Beleving van de Zwangerschapsschaal- verkorte versie’ [55]. Of the total scale, consisting of the two subscales Uplifts (positive pregnancy experiences) and Hassles (negative pregnancy experiences), only the 10-item Uplift scale will be used in this study. Examples of items are ‘How much the baby is moving’ and ‘Making or thinking about nursery arrangements’ and participants will be asked the extent to which a particular experience makes them happy, answered on a 4-item Likert scale ranging from 0 (not at all) to 4 (very much). Higher scores indicate a more positive pregnancy experience. The BZS-K Uplift scale has good internal consistency (*α* = .83) and good intraclass correlation coefficients (*ICC* = .72–.84; [55]).

##### Intrinsic motivation

Intrinsic motivation for behavior change will be asked by five questions based on questions from the Treatment Self-Regulation Questionnaire (TSRQ) [69]. Examples of questions are ‘I think that quitting smoking is best for my health’ and ‘I think that quitting smoking is best for the health of my baby’ answered on a five-point Likert scale ranging from (1 = totally disagree, 5 = totally agree). Higher scores indicate higher intrinsic motivation to quit smoking. Internal consistency of the TSRQ was found to be acceptable [69].

#### Covariates

##### Level of addiction

The ‘Heaviness of Smoking Index’ (HSI; [70]) will be used to assess the level of smoking addiction of the women. The question on the number of cigarettes smoked on a day in combination with a question asking about the time to first smoking in the morning generates the ‘Heaviness of smoking index’. The question on time to first smoking can be answered with ‘within 5 minutes’ (3 points), ‘after 6-30 minutes’ (2 points), ‘31 to 60 min’ (1 point), or ‘after 60 minutes’ (0 points). To create a total HSI score, the answer to the question about the number of cigarettes smoked on a day are categorized in four groups and each group will be given a different amount of points: 0 (10 or fewer), 1 (11 to 20), 2 (21–30), 3 (31 or more). Scores of the two questions will be summed in order to obtain three levels of addiction; low addiction (score between 0 and 2), moderate addiction (3, 4), high addiction (5, 6).

##### COVID 19-related stress

COVID-19-related concerns will be assessed using self-constructed questions. Participants will be asked to answer questions such as “Have you been infected by the COVID-19 virus?” (yes/no), “How much do you worry about the COVID-19 virus?”, and “How much do you worry about your own health and the health of your unborn child?”, which can be answered on a scale from 0 (Not worried at all) to 10 (Extremely worried). From the scale questions a sum score will be calculated with higher scores indicating more COVID-19-related concerns.

##### History of psychological complaints

History of psychological complaints will be asked using three self-constructed questions, i.e. “Did you receive treatment for psychological complaints in the past year?” (yes/no), and if so, “For which complaints have you been treated?” and “Do you still experience complaints?” (yes/no). Answers to these questions will be categorized in 0 (never received treatment), 1 (history of treatment), 2 (currently experiencing complaints).

##### Depression symptoms

Depression symptoms will be assessed using the 7-item ‘Depression’ subscale of the Depression, Anxiety, Stress Scale-21 (DASS short form; [71, 72]). Participants are asked to indicate the extent to which they had experienced specific symptoms over the previous week on a 4-point Likert scale ranging from 0 (Never) to 3 (Most of the time). Higher scores indicate higher levels of depressive symptoms. Internal consistency of the depression subscale has found to be good (Cronbach’s ﻿α = .91; [71]).

##### Reasons of smoking

Reasons of smoking will be asked with six self-constructed questions, e.g. ‘I smoke because it makes me calm’ and ‘I smoke because I am bored’ (1 = totally disagree, 5 = totally agree).

##### Birth characteristics

Complications and hospitalization during and after childbirth of the mother and child will be asked by questions such as “Did you have any complications during delivery?” and “Was your baby admitted to the hospital immediately after birth or in the first week after birth?”. When answered “yes”, questions will appear asking about the type of complications and reason for hospitalization.

In order to improve comprehension of specific parts of the questionnaires were adapted to our target group, consisting of women of lower SES. Some items under ‘Experienced stress’, ‘Depression’, ‘Intrinsic motivation’ are adapted. Also, answer categories of ‘Experienced stress’, ‘Self-efficacy regarding smoking cessation’ (0 = never to 3 = always) and ‘Perceived mother-child bond’ were reduced or adapted to increase consistency among questionnaires and to improve clarification.

### Statistical analyses

**Fig. 3:**
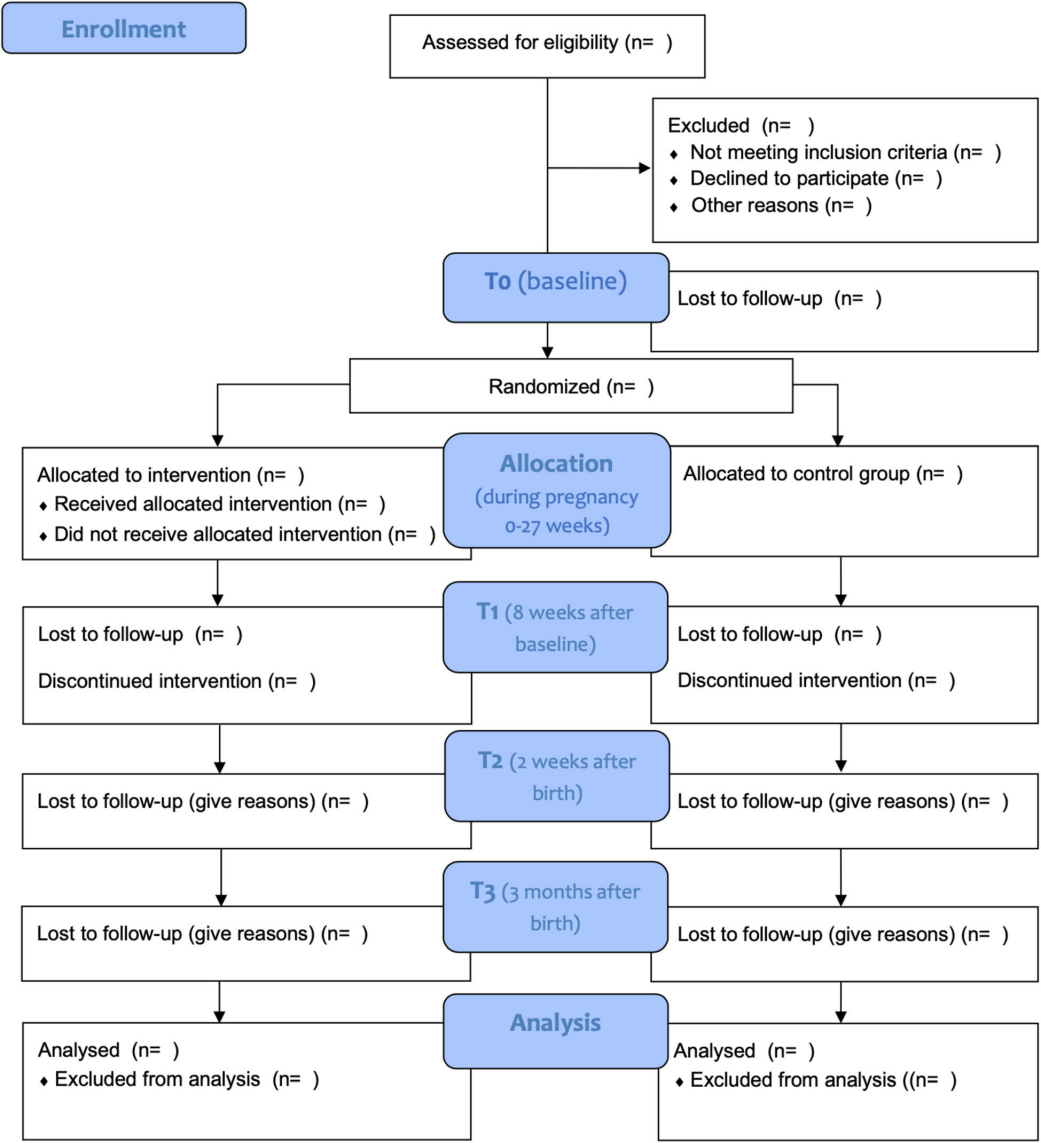
Consolidated Standards of Reporting Trials (CONSORT) flow diagram

The study will be performed according to the Consolidated Standards of Reporting Trials (CONSORT) Statement (see Fig. 3). All analysis will be done in SPSS version 26.0 and ﻿RStudio version 1.1.456 and a significance level of 0.05 will be used. Analyses will be performed conforming to intention-to-treat analysis, so that all randomized participants will be analyzed. We will explore baseline data to examine differences between the two different conditions and between the study completers and drop-outs. We will control for relevant covariates (i.e. SES, treatment history, maternal age, gestational age at birth, level of addiction, birth complications, way of delivery) by adding them to the models. Prior to the main analyses, outliers will be checked. Demographic outcomes will be compared using chi-square tests and independent sample t-tests.

To examine the effectiveness of the eHealth intervention on the primary outcome, the number of women who quit smoking during pregnancy measured at post-intervention and follow-up, General Estimating Equations (GEE) using a logistic model will be performed. Potential covariates that will be included in the model are maternal age, depressive symptoms, gestational age, employment status, education level, level of addiction, and partner’s smoking status. To investigate the effectiveness of the intervention on secondary outcomes (i.e., smoking behavior, perceived stress, birth outcomes, negative affectivity, self-efficacy, mother-child bonding, intrinsic motivation, parenting self-efficacy, pregnancy-related anxiety) linear mixed models will be performed for each outcome variable separately. Furthermore, we will test whether the effect of the intervention (independent variable) on smoking cessation (dependent variable) is mediated by a change in perceived stress using a GEE model. Moreover, it will be tested whether program usage and experience, self-efficacy regarding smoking cessation, feelings of mother-child bonding, intrinsic motivation for life style behavior change, and pregnancy experience moderate the effect on smoking cessation (primary outcome), smoking behaviour, and perceived stress by adding the variables to the GEE model.

### Patient and public involvement

The design of the content of both the eHealth intervention and the control condition was achieved by co-creating processes with focus groups of women with low socioeconomic backgrounds, who either successfully quit smoking or struggled with quitting smoking during pregnancy. Data collection is carried out in collaboration with midwives and nurses from various midwifery practices and hospitals throughout the Netherlands.

## Discussion

The objective of this study is to examine the effectiveness of an eHealth intervention to help pregnant smoking women quit smoking. In contrast to other smoking-cessation interventions, this app-intervention particularly focuses on teaching women new stress-coping techniques. Such a component has been missing in existing stop-smoking programs. Stress is a common factor among smokers that hampers quitting and may result in relapse, in particular for women of low socioeconomic backgrounds**.** Smoking is a common behavior to alleviate feelings of tension. Despite this co-occurrence of smoking with high levels of stress among low SES pregnant women, we still lack an intervention that also supports pregnant women in learning new stress-coping skills. Learning new stress-coping skills might not only help these women quit smoking during their pregnancy, but can also help women stay abstinent after the birth of their baby.

‘Together with Eva’ is developed for women with low SES by means of easy and understandable language, attractive design, many repetitions of both text and exercises, positive feedback, and instruction videos of the different components of the application (i.e. stop-smoking plan, diary, breathing exercises). This was done in co-creation with women from the target group. Thereby, we expect to achieve the most beneficial results for this group of smoking pregnant women. As, once developed, the intervention can be made available to users for a low price, is easily accessible, and easy to implement into the health care system, we expect that this app-intervention will be a useful tool both for health care professionals to offer to their patients and for the pregnant women to receive support with quitting smoking during pregnancy. All in all, a personalized intervention for this target group is needed to further reduce inequalities in perinatal and maternal outcomes in in high income countries.

### Status of the trial

After receiving permission by the Medical Ethics Committee to start with the inclusion of participants, the first participants were included in July 2020. Currently, data collection is ongoing. The main results are expected to be published in 2022.

## Data Availability

The datasets generated and/or analyzed during the current study will be available from the corresponding author on reasonable request.

## Abbreviations

ACT: Acceptance and Commitment Therapy
BZS: Beleving van de Zwangerschap Schaal
CBT: Cognitive Behavior Therapy
CONSORT: Consolidated Standards of Reporting Trials
DASS: Depression, Anxiety, Stress Scale
HRV: Heart Rate Variability
HRV-BF: Heart Rate Variability Bio-feedback
HSI: Heaviness of Smoking Index
IBQ: Infant Behavior Questionnaire
METc: Medical Ethics Committee
NTR: Netherlands Trial Register
PES: Pregnancy Experience Scale
PRAQ: Pregnancy-related Anxiety Questionnaire
PSS: Perceived Stress Scale
RCT: Randomized Controlled Trial
SDK: Software Developer Kit
SENR: Self-efficacy in the Nurturing Role questionnaire
SES: Socio-economic status
SSFSL: Stress- and Smoke Free Start of Life
TSRQ: Treatment Self-regulation Questionnaire
t0: baseline assessment
t1: second assessment
t2: third assessment (first follow-up)
t3: fourth assessment (second follow-up)
UEQ: User Experience Questionnaire
VUmc: VU University Medical Center
